# Independent prognostic impact of DNA methylation class and chromosome 1p loss in WHO grade 2 and 3 meningioma undergoing adjuvant high-dose radiotherapy: comprehensive molecular analysis of EORTC 22042–26042

**DOI:** 10.1007/s00401-023-02642-5

**Published:** 2023-10-19

**Authors:** Sybren L. N. Maas, Philipp Sievers, Damien C. Weber, Michael Weller, Martin J. van den Bent, Maximilian J. Mair, Johan M. Kros, Fransesca Carparrotti, Andreas von Deimling, Villà Freixa Salvador, Saskia Marguerite Peerdeman, Jose Casas-Martin, Thierry Gorlia, Felix Sahm, Matthias Preusser

**Affiliations:** 1 Department of Pathology, Leiden University Medical Center, Leiden, The Netherlands; 2 Department of Pathology, Erasmus MC Cancer Institute, University Medical Center Rotterdam, Rotterdam, The Netherlands; 3 Department of Neuropathology, Institute of Pathology, Ruprecht-Karls-University Heidelberg, Heidelberg, Germany; 4 Paul Scherrer Institute, Villigen PSI, Switzerland; 5 Department of Radiation Oncology, University Hospital and University of Zurich, Zurich, Switzerland; 6 Department of Neurology, Clinical Neuroscience Center, University Hospital and University of Zurich, Zurich, Switzerland; 7 The Brain Tumor Center, Erasmus Medical Center Cancer Institute, Rotterdam, The Netherlands; 8 Division of Oncology, Department of Medicine I, Medical University of Vienna, Vienna, Austria; 9 Hôpitaux Universitaires de Genève, Geneva, Switzerland; 10 ICO Badalona, Hospital Germans Trias I Pujol (Institut Catala D’Oncologia), Catalonia, Spain; 11 Department of Neurosurgery, Amsterdam UMC, Amsterdam, The Netherlands; 12 EORTC Head Quarters, Brussels, Belgium; 13 CCU Neuropathology, German Consortium for Translational Cancer Research (DKTK), German Cancer Research Center (DKFZ), Heidelberg, Germany

In the recent 2021 CNS5 WHO classification, molecular alterations were introduced into meningioma grading [4]. Specifically, mutations in the promotor area of the telomerase reverse transcriptase (*TERT*) gene and/or homozygous loss of the *CDKN2A/B* locus automatically result in a grade 3 diagnosis [3, 4]. Simultaneously meningioma with *TRAF7*, *AKT1*, *KLF4*, and/or *SMO* mutations are, in general, associated with lower progression risk [1, 10]. Alterations linked with increased risk include *BAP1* mutations [9], copy-number variations of chromosomal arms such as 1p, 6q, 10q and 14q [6], genome-wide epigenetic profiles also referred to as DNA methylation classes (MC) [8], or the combination of molecular alterations included in an integrated risk score, grade or classification [2, 5, 7]. As most studies identifying molecular risk factors are retrospective in design, independent validation is needed to further advance the adoption of meningioma molecularly based risk prediction. Here, we present additional retrospective analysis on the data derived from the European Organisation for Research and Treatment of Cancer (EORTC) 22042-26042 clinical trial, designed to prospectively evaluate the effectiveness and adverse effects of high-dose radiotherapy in the treatment of atypical (WHO grade 2) and malignant (WHO grade 3) meningiomas [11].

The EORTC 22042–26042 trial included 78 patients (69 WHO grade 2 and nine grade 3 meningioma) with different arms based on the Simpson grade, a clinical variable for the extent of the resection. Results from the primary aim of the study have been published before and identified that the 3-year progression free survival (PFS) in patients with WHO grade 2 meningioma undergoing high-dose (60 Gy) radiotherapy is significantly higher than the hypothesized primary study endpoint (88% observed versus 70% hypothesized) [11]. In the present study we screened mutations, copy-number variations and DNA methylation profiles, for their prognostic value. Of the 78 patients enrolled in the trial, 53 patients consented their resected material to be available for further research. The samples were analyzed using next generation sequencing (NGS) for (hotspot) alterations in *AKT1, TRAF7, SMO, KLF4, BAP1* and the *TERT-promotor,* for DNA methylation profiling and copy number analysis for chromosomal losses of 1p and 22q. Cases were identified as either *NF2*-type or non-NF2-type based on mutations detected in *NF2*, *AKT1, SMO, KLF4* or *TRAF7*. For *n* = 38 patients, adequate material was available for DNA methylation profiling (Fig. 1a). All molecular analyses were performed at the Department of Neuropathology of the University Hospital Heidelberg (Germany) and in-depth descriptions of the materials and methods used have been published before [5, 8]. PFS was defined as the time between the date of registration to tumor growth or death of any cause, whereas overall survival (OS) was defined as the time between date of registration and death of any cause. When comparing clinical characteristics between consensus for further research state, encompassing factors such as age, sex, performance status, and tumor attributes such as WHO grade, tumor volume, location, and Simpson grade, only the distribution of WHO grade exhibited a statistically significant difference. Notably, the group that provided consent for further analyses contained a higher proportion of patients with WHO grade 2 tumors (Supp. Table 1). Out of the *n* = 53 tumors analyzed, *SMO* mutations were not detected, *AKT1, KLF4* and *BAP1* mutations were detected once, *TERT*-promotor mutations twice and a total of three cases with mutations in *TRAF7,* hampering further analyses of prognostic value of these markers (Suppl. Table 2). Deletions of *CDKN2A/B* were detected three times (2 homozygous losses and 1 heterozygous loss), 1p loss 23 times (60.5%) and 22q losses 29 times (76.3%). The frequency of observed events was generally similar to larger meningioma cohorts [10]. Most cases were identified as intermediate meningioma MC (44.7%), followed by malignant (28.9%) and benign (26.3%) MC (molecular events per MC in Suppl. Table 3). These three overarching MC can be subdivided into six subclasses (benign-1, benign-2, benign-3, intermediate-A, intermediate-B and malignant) but due to the relatively small number of cases included, further analyses only included the three main MCs benign, intermediate and malignant [8]. Since the aim of this study was to identify molecular markers associated with risk for progression and only 3 WHO grade 3 cases with consent were available, the two WHO grade groups were aggregated for further analysis.

**Fig. 1:**
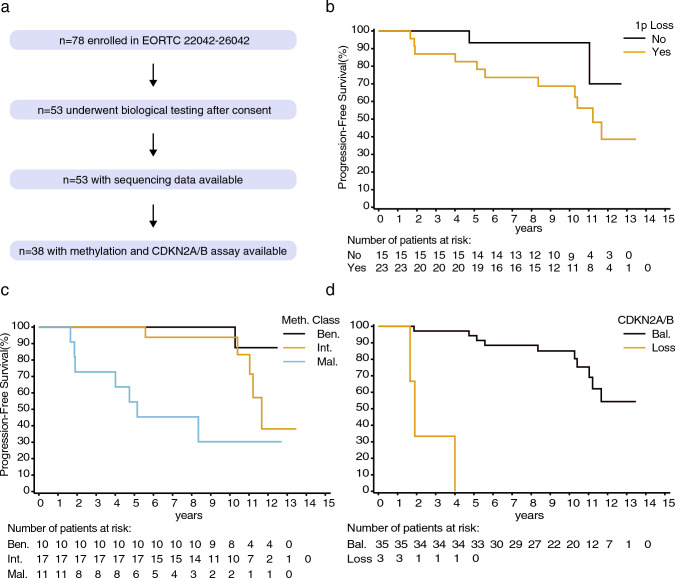
Graphical illustration of molecular data availability among EORTC 22042-26042 trial patients (**a**). Kaplan–Meier plots showing the impact of chromosome 1p (**b**), methylation class (**c**) and *CDKN2A/B* loss on progression-free survival (**d**)

PFS univariate analysis at 10% significance identified sex, Simpson stage, age (median), 1p loss (Fig. 1b), meningioma MC (Fig. 1c) and *CDKN2A/B* loss (Fig. 1d) as prognostic factors (Suppl. Table 4). In a subset of cases with residual tumor (*n* = 24), all volumetric measurements above the median, except for tumor length, were significantly associated with worse PFS. Due to the low number of cases with residual tumor and adequate molecular information available (*n* = 11), or a small number of events for *CDKN2A/B*, further multivariate analyses on these (volumetric) measurements could not be performed. In the final multivariate clinical and molecular full Cox model, loss of chromosome 1p and MC were identified as significant factors at the 10% significance level (Suppl. Table 5).

OS univariate analysis at 10% significance identified WHO performance status, sex, Simpson stage, age (median), MC and *CDKN2A/B* loss as prognostic factors (Suppl. Table 6). In the multivariate clinical and molecular full Cox model however, none of the factors reached significance, possibly due to the limited number of OS events available (*e* = 8) compared to the total number of modalities included in the model (*k* = 7; Suppl. Table 7).

In conclusion, this study identifies independent prognostic impact of MC and 1p loss on PFS in a cohort of atypical and malignant meningioma undergoing high-dose radiotherapy. In line, 1p loss is observed in most meningioma of (epi)genetically and/or transcriptomically defined increased risk [6]. Hence, 1p analysis may provide an independent, cost-efficient marker for identification of cases at higher risk of recurrence. Due to the relatively small cohort and events, the association of *CDKN2A/B* loss and *TERT-*promotor mutations with outcome could not be validated and should be investigated in a larger prospective cohort with longer follow-up.

### Supplementary Information

Below is the link to the electronic supplementary material.

Supplementary file1 (DOCX 59 KB)

## Data Availability

All data sharing requests should be directed to the EORTC and will be evaluated according to standard operating procedures of the EORTC Brain Tumor Group. Data sharing requires compliance with current data protection regulations by the European Union, a positive vote by competent ethics committee, and data access agreements with the EORTC.
