# Supplementary material for: Independent prognostic impact of DNA methylation class and chromosome 1p loss in WHO grade 2 and 3 meningioma undergoing adjuvant high-dose radiotherapy: comprehensive molecular analysis of EORTC 22042–26042
Source: Acta Neuropathol. 2023 Oct 19;146(6):837–40. doi: 10.1007/s00401-023-02642-5 (PMC10627973; doi:10.1007/s00401-023-02642-5)
Supplement: Supplementary file 1 — Supplementary file1 (DOCX 59 KB) [file 401_2023_2642_MOESM1_ESM.docx]

**Suppl. Table 1**: clinical data split on consent for molecular testing

|  | **With (consented) genetic results** | | **Total (N=78)** |  |
| --- | --- | --- | --- | --- |
|  | **No (N=25)** | **Yes (N=53)** |  | P |
|  | **N (%)** | **N (%)** | **N (%)** |  |
| **Performance status** |  |  |  | 0.88 |
| N obs | 25 (100.0) | 53 (100.0) | 78 (100.0) |  |
| 0 | 17 (68.0) | 38 (71.7) | 55 (70.5) |  |
| 1 | 6 (24.0) | 8 (15.1) | 14 (17.9) |  |
| 2 | 2 (8.0) | 7 (13.2) | 9 (11.5) |  |
| **Sex** |  |  |  | 0.81 |
| N obs | 25 (100.0) | 53 (100.0) | 78 (100.0) |  |
| male | 14 (56.0) | 27 (50.9) | 41 (52.6) |  |
| female | 11 (44.0) | 26 (49.1) | 37 (47.4) |  |
| **Age** |  |  |  | 0.69 |
| Median | 53.0 | 54.0 | 54.0 |  |
| Range | 29.0 - 69.0 | 21.0 - 72.0 | 21.0 - 72.0 |  |
| Mean (SD) | 52.08 (11.91) | 53.23 (11.49) | 52.86 (11.56) |  |
| N obs | 25 | 53 | 78 |  |
| **Mini Mental State Evaluation** |  |  |  | 0.07 |
| Median | 30.0 | 29.0 | 29.0 |  |
| Range | 26.0 - 30.0 | 20.0 - 30.0 | 20.0 - 30.0 |  |
| Mean (SD) | 29.26 (1.10) | 28.06 (2.49) | 28.43 (2.22) |  |
| N obs | 23 | 51 | 74 |  |
| **MMSE at baseline** |  |  |  | 0.05 |
| N obs | 23 (92.0) | 51 (96.2) | 74 (94.9) |  |
| score <= 26 | 1 (4.3) | 12 (23.5) | 13 (17.6) |  |
| score >= 27 | 22 (95.7) | 39 (76.5) | 61 (82.4) |  |
| **Time from surgery to registration (months)** |  |  |  | 0.12 |
| Median | 1.2 | 1.0 | 1.1 |  |
| Range | 0.6 - 2.0 | 0.4 - 1.9 | 0.4 - 2.0 |  |
| Mean (SD) | 1.20 (0.34) | 1.07 (0.35) | 1.11 (0.35) |  |
| N obs | 25 | 53 | 78 |  |
| **Time from post-surgery MRI to registration (months)*** |  |  |  | 0.11 |
| Median | 0.6 | 0.4 | 0.4 |  |
| Range | -0.2 - 1.5 | -0.2 - 1.8 | -0.2 - 1.8 |  |
| Mean (SD) | 0.65 (0.46) | 0.51 (0.47) | 0.56 (0.47) |  |
| N obs | 24 | 53 | 77 |  |
| **WHO grade** |  |  |  | 0.03 |
| N obs | 25 (100.0) | 53 (100.0) | 78 (100.0) |  |
| 2 | 19 (76.0) | 50 (94.3) | 69 (88.5) |  |
| 3 | 6 (24.0) | 3 (5.7) | 9 (11.5) |  |
| **Simpson's stage** |  |  |  | 0.64 |
| N obs | 25 (100.0) | 53 (100.0) | 78 (100.0) |  |
| 1 | 5 (20.0) | 11 (20.8) | 16 (20.5) |  |
| 2 | 8 (32.0) | 21 (39.6) | 29 (37.2) |  |
| 3 | 7 (28.0) | 12 (22.6) | 19 (24.4) |  |
| 4 | 5 (20.0) | 8 (15.1) | 13 (16.7) |  |
| 5 | 0 (0.0) | 1 (1.9) | 1 (1.3) |  |
| **Tumor location** |  |  |  | 0.95 |
| N obs | 25 (100.0) | 53 (100.0) | 78 (100.0) |  |
| Olfact groove | 0 (0.0) | 1 (1.9) | 1 (1.3) |  |
| Medial Sphenoid wing/parasellar | 2 (8.0) | 2 (3.8) | 4 (5.1) |  |
| Tentorial | 0 (0.0) | 1 (1.9) | 1 (1.3) |  |
| Cerebellopontine angle | 1 (4.0) | 1 (1.9) | 2 (2.6) |  |
| Falx/parasagittal | 6 (24.0) | 12 (22.6) | 18 (23.1) |  |
| Convexity | 10 (40.0) | 23 (43.4) | 33 (42.3) |  |
| Other | 6 (24.0) | 13 (24.5) | 19 (24.4) |  |
| **Tumor location** |  |  |  | 1.0 |
| N obs | 25 (100.0) | 53 (100.0) | 78 (100.0) |  |
| Skull base | 3 (12.0) | 7 (13.2) | 10 (12.8) |  |
| Convexity | 22 (88.0) | 46 (86.8) | 68 (87.2) |  |
| **Residual tumor** |  |  |  | 0.59 |
| N obs | 23 (92.0) | 51 (96.2) | 74 (94.9) |  |
| No residual tumor | 17 (73.9) | 33 (64.7) | 50 (67.6) |  |
| Residual tumor | 6 (26.1) | 18 (35.3) | 24 (32.4) |  |
| **Corticosteroids** |  |  |  | 1.0 |
| N obs | 25 (100.0) | 53 (100.0) | 78 (100.0) |  |
| no | 18 (72.0) | 39 (73.6) | 57 (73.1) |  |
| yes | 7 (28.0) | 14 (26.4) | 21 (26.9) |  |
| **Dexamethasone** | 5 (20.0) | 13 (24.5) | 18 (23.1) |  |
| **Methylprednisolone** | 0 (0.0) | 1 (1.9) | 1 (1.3) |  |
| **other** | 2 (8.0) | 0 (0.0) | 2 (2.6) |  |
| **Anti-epileptic treatment** |  |  |  | 1.0 |
| N obs | 25 (100.0) | 52 (98.1) | 77 (98.7) |  |
| no | 12 (48.0) | 24 (46.2) | 36 (46.8) |  |
| yes | 13 (52.0) | 28 (53.8) | 41 (53.2) |  |
| **EIAED** | 3 (12.0) | 5 (9.4) | 8 (10.3) |  |
| other | 10 (40.0) | 23 (43.4) | 33 (42.3) |  |

**Suppl. Table 2**: molecular data for patient that consented to testing

|  | **With (consented) genetic results** |
| --- | --- |
|  | **Yes (N=53)** |
|  | **N (%)** |
| **AKT1 mutational status** |  |
| N obs | 53 (100.0) |
| No | 52 (98.1) |
| Yes | 1 (1.9) |
| **BAP1 mutational status** |  |
| N obs | 53 (100.0) |
| No | 52 (98.1) |
| Yes | 1 (1.9) |
| **NF2 type** |  |
| N obs | 53 (100.0) |
| No | 27 (50.9) |
| Yes | 26 (49.1) |
| **TERT promoter mutational status** |  |
| N obs | 53 (100.0) |
| No | 51 (96.2) |
| Yes | 2 (3.8) |
| **TRAF7 mutational status** |  |
| N obs | 53 (100.0) |
| No | 50 (94.3) |
| Yes | 3 (5.7) |
| **KLF4 mutational status** |  |
| N obs | 53 (100.0) |
| No | 52 (98.1) |
| Yes | 1 (1.9) |
| **SMO mutational status** |  |
| N obs | 53 (100.0) |
| No | 53 (100.0) |
| Yes | 0 (0.0) |
| **CDKNA2A/B deletion status** |  |
| N obs | 38 (71.7) |
| Balanced | 35 (92.1) |
| Homozygous deletion | 2 (5.3) |
| Heterozygous deletion | 1 (2.6) |
| **Methylation class (MC)** |  |
| N ob | 38 (71.7) |
| ben-1 | 3 (7.9) |
| ben-2 | 5 (13.2) |
| ben-3 | 2 (5.3) |
| int-A | 15 (39.5) |
| int-B | 2 (5.3) |
| mal | 11 (28.9) |
| **Methylation class family** |  |
| N obs | 38 (71.7) |
| benign | 10 (26.3) |
| intermediate | 17 (44.7) |
| malignant | 11 (28.9) |

**Suppl. Table 3**: distribution of molecular alterations over the methylation classes

|  | **Meningioma class** | | | | **Total (N=78)** |  |
| --- | --- | --- | --- | --- | --- | --- |
|  | **Unknown (N=40)** | **benign (N=10)** | **intermediate (N=17)** | **malignant (N=11)** |  | P |
|  | **N (%)** | **N (%)** | **N (%)** | **N (%)** | **N (%)** |  |
| **AKT1 mutational status** |  |  |  |  |  | 0.24 |
| **N obs** | 15 (37.5) | 10 (100.0) | 17 (100.0) | 11 (100.0) | 53 (67.9) |  |
| **Normal** | 15 (100.0) | 9 (90.0) | 17 (100.0) | 11 (100.0) | 52 (98.1) |  |
| **Mutated** | 0 (0.0) | 1 (10.0) | 0 (0.0) | 0 (0.0) | 1 (1.9) |  |
| **BAP1 mutational status** |  |  |  |  |  | 0.54 |
| **N obs** | 15 (37.5) | 10 (100.0) | 17 (100.0) | 11 (100.0) | 53 (67.9) |  |
| **Normal** | 15 (100.0) | 10 (100.0) | 17 (100.0) | 10 (90.9) | 52 (98.1) |  |
| **Mutated** | 0 (0.0) | 0 (0.0) | 0 (0.0) | 1 (9.1) | 1 (1.9) |  |
| **NF2 type** |  |  |  |  |  | 0.39 |
| **N obs** | 15 (37.5) | 10 (100.0) | 17 (100.0) | 11 (100.0) | 53 (67.9) |  |
| **Normal** | 7 (46.7) | 7 (70.0) | 7 (41.2) | 6 (54.5) | 27 (50.9) |  |
| **Mutated** | 8 (53.3) | 3 (30.0) | 10 (58.8) | 5 (45.5) | 26 (49.1) |  |
| **TERT promoter mutational status** |  |  |  |  |  | 0.55 |
| **N obs** | 15 (37.5) | 10 (100.0) | 17 (100.0) | 11 (100.0) | 53 (67.9) |  |
| **Normal** | 14 (93.3) | 10 (100.0) | 17 (100.0) | 10 (90.9) | 51 (96.2) |  |
| **Mutated** | 1 (6.7) | 0 (0.0) | 0 (0.0) | 1 (9.1) | 2 (3.8) |  |
| **1p loss** |  |  |  |  |  | 0.34 |
| **N obs** | N/A | 10 (100.0) | 17 (100.0) | 11 (100.0) | 38 (48.7) |  |
| **No** | N/A | 6 (60.0) | 5 (29.4) | 4 (36.4) | 15 (39.5) |  |
| **Yes** | N/A | 4 (40.0) | 12 (70.6) | 7 (63.6) | 23 (60.5) |  |
| **22q loss** |  |  |  |  |  | 0.03 |
| **N obs** | N/A | 10 (100.0) | 17 (100.0) | 11 (100.0) | 38 (48.7) |  |
| **No** | N/A | 5 (50.0) | 1 (5.9) | 3 (27.3) | 9 (23.7) |  |
| **Yes** | N/A | 5 (50.0) | 16 (94.1) | 8 (72.7) | 29 (76.3) |  |
| **CDKN2AB** |  |  |  |  |  | 0.03 |
| **N obs** | N/A | 10 (100.0) | 17 (100.0) | 11 (100.0) | 38 (48.7) |  |
| **Balanced** | N/A | 10 (100.0) | 17 (100.0) | 8 (72.7) | 35 (92.1) |  |
| **Del** | N/A | 0 (0.0) | 0 (0.0) | 3 (27.3) | 3 (7.9) |  |

**Suppl. Table 4**: Univariate testing for PFS

|  | **Progression-Free Survival** | | | | | |
| --- | --- | --- | --- | --- | --- | --- |
|  | | **Event/Total** | **Median (95% CI) (Years)**^KM^ | **3 years Survival Estimates (95% CI)**^KM^ | **Hazard Ratio (90% CI)**^Cox^ | **P-Value (Logrank)** |
| **Performance status** | |  |  |  |  | 0.1923^*^ |
| 0 | | 21/55 | 11.2 (9.8-NE) | 86.8 (74.2-93.5%) | Reference |  |
| 1 | | 5/14 | NE (5.6-NE) | 100.0 (100.0-100.0%) | 0.99 (0.44-2.27) |  |
| 2 | | 1/9 | NE (11.0-NE) | 100.0 (100.0-100.0%) | 0.19 (0.03-1.02) |  |
| **Sex** | |  |  |  |  | 0.0524^*^ |
| female | | 9/37 | NE (11.0-NE) | 97.1 (81.4-99.6%) | Reference |  |
| male | | 18/41 | 10.4 (8.4-NE) | 84.9 (69.5-92.9%) | 2.17 (1.11-4.25) |  |
| **MMSE at baseline** | |  |  |  |  | 0.2789^*^ |
| score <= 26 | | 6/13 | 11.0 (1.9-NE) | 83.3 (48.2-95.6%) | Reference |  |
| score >= 27 | | 20/61 | 11.7 (10.3-NE) | 91.6 (81.1-96.4%) | 0.61 (0.28-1.31) |  |
| **WHO grade** | |  |  |  |  | 0.4324^*^ |
| 2 | | 23/69 | 11.7 (10.4-NE) | 90.9 (80.9-95.8%) | Reference |  |
| 3 | | 4/9 | NE (1.7-NE) | 88.9 (43.3-98.4%) | 1.53 (0.62-3.75) |  |
| **Residual tumor** | |  |  |  |  | 0.2901* |
| No residual tumor | | 17/50 | 11.7 (10.4-NE) | 91.8 (79.7-96.9%) | Reference |  |
| Residual tumor | | 9/24 | NE (5.1-NE) | 86.4 (63.4-95.4%) | 1.55 (0.78-3.08) |  |
| **Simpson’ stage/treatment** | |  |  |  |  | 0.0425^*^ |
| 1-3 | | 20/64 | 11.7 (11.0-NE) | 90.3 (79.7-95.5%) | Reference |  |
| 4-5 | | 7/14 | 9.0 (4.7-NE) | 92.3 (56.6-98.9%) | 2.42 (1.16-5.05) |  |
| **Corticosteroids** | |  |  |  |  | 0.7029^*^ |
| no | | 20/57 | 11.2 (10.3-NE) | 89.3 (77.7-95.0%) | Reference |  |
| yes | | 7/21 | 11.7 (9.0-NE) | 94.7 (68.1-99.2%) | 0.85 (0.41-1.75) |  |
| **Anti-epileptic treatment** | |  |  |  |  | 0.9454^*^ |
| no | | 12/36 | NE (9.8-NE) | 91.2 (75.1-97.1%) | Reference |  |
| yes | | 14/41 | 11.7 (10.4-NE) | 92.4 (78.3-97.5%) | 0.97 (0.51-1.86) |  |
| **Age (median = 54y)** | |  |  |  |  | 0.0717^*^ |
| <=median | | 9/37 | NE (10.3-NE) | 91.6 (76.1-97.2%) | Reference |  |
| >median | | 18/41 | 11.0 (8.4-NE) | 89.7 (74.9-96.0%) | 2.06 (1.05-4.04) |  |
| **Initial tumour measurement - length (median = 20.5 mm)*** | |  |  |  |  | 0.3926^*^ |
| <=median | | 3/12 | NE (0.9-NE) | 90.0 (47.3-98.5%) | Reference |  |
| >median | | 6/12 | 8.4 (2.9-NE) | 83.3 (48.2-95.6%) | 1.82 (0.57-5.83) |  |
| **Initial tumour measurement - width (median = 15 mm)*** | |  |  |  |  | 0.0403^*^ |
| <=median | | 1/11 | NE (6.2-NE) | 100.0 (100.0-100.0%) | Reference |  |
| >median | | 7/12 | 7.7 (1.9-NE) | 75.0 (40.8-91.2%) | 6.69 (1.15-38.96) |  |
| **Initial tumour measurement - height (median = 11.5 mm)*** | |  |  |  |  | 0.0147^*^ |
| <=median | | 1/11 | NE (6.2-NE) | 100.0 (100.0-100.0%) | Reference |  |
| >median | | 7/11 | 7.0 (1.9-NE) | 72.7 (37.1-90.3%) | 8.85 (1.51-51.81) |  |
| **Product of measurements by DM (median = 2721 mm³)*** | |  |  |  |  | 0.0225^*^ |
| <=median | | 1/11 | NE (6.2-NE) | 100.0 (100.0-100.0%) | Reference |  |
| >median | | 7/11 | 7.0 (1.9-NE) | 72.7 (37.1-90.3%) | 7.88 (1.35-45.89) |  |
| **Intital volume (median = 3126.9 mm³)*** | |  |  |  |  | 0.0225^*^ |
| <=median | | 1/11 | NE (6.2-NE) | 100.0 (100.0-100.0%) | Reference |  |
| >median | | 7/11 | 7.0 (1.9-NE) | 72.7 (37.1-90.3%) | 7.88 (1.35-45.89) |  |
| **Initial maximum diameter (median = 24.5 mm)*** | |  |  |  |  | 0.0956^*^ |
| <=median | | 2/12 | NE (3.9-NE) | 100.0 (100.0-100.0%) | Reference |  |
| >median | | 7/12 | 7.7 (1.9-NE) | 75.0 (40.8-91.2%) | 3.51 (0.94-13.15) |  |
| **NF2 type**** | |  |  |  |  | 0.4999^*^ |
| No | | 8/27 | NE (10.3-NE) | 96.3 (76.5-99.5%) | Reference |  |
| Yes | | 9/26 | 11.7 (10.4-NE) | 88.0 (67.3-96.0%) | 1.39 (0.62-3.09) |  |
| **1p loss**** | |  |  |  |  | 0.0724^*^ |
| No | | 2/15 | NE (11.0-NE) | 100.0 (100.0-100.0%) | Reference |  |
| Yes | | 11/23 | 11.2 (8.4-NE) | 87.0 (64.8-95.6%) | 3.64 (1.03-12.93) |  |
| **22q loss**** | |  |  |  |  | 0.3996^*^ |
| No | | 2/9 | NE (4.7-NE) | 100.0 (100.0-100.0%) | Reference |  |
| Yes | | 11/29 | 11.7 (10.4-NE) | 89.7 (71.3-96.5%) | 1.89 (0.53-6.74) |  |
| **CDKN2AB**** | |  |  |  |  | <.0001^*^ |
| Balanced | | 10/35 | NE (11.0-NE) | 97.1 (81.4-99.6%) | Reference |  |
| Deleted | | 3/3 | 1.9 (1.7-NE) | 33.3 (0.9-77.4%) | 57.64 (8.38-396.25) |  |
| **Meningioma class**** | |  |  |  |  | 0.0006^*^ |
| benign | | 1/10 | NE (10.3-NE) | 100.0 (100.0-100.0%) | Reference |  |
| intermediate | | 5/17 | 11.7 (10.4-NE) | 100.0 (100.0-100.0%) | 3.53 (0.58-21.49) |  |
| malignant | | 7/11 | 5.1 (1.9-NE) | 72.7 (37.1-90.3%) | 17.05 (2.86-101.78) |  |
|  | ^KM^Kaplan-Meier method; ^Cox^Cox model; ^*^Logrank test; | | | | | |

Note: Significant associations (p<0.10) are shaded in grey. * Initial measurements analyses including the assessment of the median were performed in the patients with residual disease and available measurement data (n=24). Clinical data were assessed in the ITT population (n=78). **Molecular data were assessed in available tissues of patients consenting to biological research (n=53 for NF2 type and n=38 for 1p loss,22q loss, CDKN2AB, Meningioma class). AKT1 (1 mutated), BAP1 (1 mutated), TERTp (2 mutated), TRAF7 had very small number of molecular events and were not assessed.

**Suppl. Table 5**: Multivariate testing for PFS

| **Parameter** |  | **DF** | **Parameter Estimate** | **Standard Error** | **Chi-Square** | **Pr > ChiSq** | **Hazard Ratio** | **90% Hazard Ratio CL** | |  |
| --- | --- | --- | --- | --- | --- | --- | --- | --- | --- | --- |
| **Sex** | **Male** | 1 | 0.37995 | 0.66997 | 0.3216 | 0.5706 | 1.462 | 0.486 | 4.402 | |
| **Simpson’ stage/treatment** | **4-5** | 1 | 0.66366 | 0.73208 | 0.8218 | 0.3646 | 1.942 | 0.582 | 6.474 | |
| **Age** | **>median** | 1 | 0.74972 | 0.72338 | 1.0741 | 0.3000 | 2.116 | 0.644 | 6.956 | |
| **1p loss** | **Yes** | 1 | 1.67231 | 0.86624 | 3.7270 | **0.0535** | **5.324** | **1.281** | **22.134** | |
| **Meningioma class** | **intermediate** | 1 | 0.39136 | 1.21341 | 0.1040 | 0.7471 | 1.479 | 0.201 | 10.884 | |
|  | **malignant** | 1 | 2.76447 | 1.25748 | 4.8331 | **0.0279** | **15.871** | **2.006** | **125.566** | |

**Suppl. Table 6**: Univariate testing for OS

|  | **OS** | | | | | |
| --- | --- | --- | --- | --- | --- | --- |
|  | | **Event/Total** | **Median (95% CI) (Years)**^KM^ | **3 years Survival Estimates (95% CI)**^KM^ | **Hazard Ratio (90% CI)**^Cox^ | **P-Value (Logrank)** |
| **Performance status** | |  |  |  |  | 0.0909^*^ |
| 0 | | 17/55 | NE (10.2-NE) | 98.1 (87.4-99.7%) | Reference |  |
| 1 | | 1/14 | NE (NE-NE) | 100.0 (100.0-100.0%) | 0.19 (0.04-1.05) |  |
| 2 | | 1/9 | NE (11.0-NE) | 100.0 (100.0-100.0%) | 0.26 (0.05-1.45) |  |
| **Sex** | |  |  |  |  | 0.0994^*^ |
| Female | | 6/37 | NE (11.0-NE) | 100.0 (100.0-100.0%) | Reference |  |
| Male | | 13/41 | NE (10.2-NE) | 97.5 (83.5-99.6%) | 2.21 (0.98-4.99) |  |
| **MMSE at baseline** | |  |  |  |  | 0.2539^*^ |
| score <= 26 | | 5/13 | 11.0 (7.0-NE) | 91.7 (53.9-98.8%) | Reference |  |
| score >= 27 | | 14/61 | NE (NE-NE) | 100.0 (100.0-100.0%) | 0.56 (0.24-1.31) |  |
| **WHO grade** | |  |  |  |  | 0.0726^*^ |
| 2 | | 15/69 | NE (NE-NE) | 98.5 (89.7-99.8%) | Reference |  |
| 3 | | 4/9 | NE (3.9-NE) | 100.0 (100.0-100.0%) | 2.68 (1.05-6.85) |  |
| **Residual tumor** | |  |  |  |  | 0.4515* |
| No residual tumor | | 12/50 | NE (11.0-NE) | 100.0 (100.0-100.0%) | Reference |  |
| Residual tumor | | 6/24 | NE (7.4-NE) | 95.5 (71.9-99.3%) | 1.46 (0.64-3.34) |  |
| **Simpson’ stage** | |  |  |  |  | 0.1080^*^ |
| 1-3 | | 14/64 | NE (11.0-NE) | 98.4 (89.1-99.8%) | Reference |  |
| 4-5 | | 5/14 | NE (7.0-NE) | 100.0 (100.0-100.0%) | 2.28 (0.96-5.43) |  |
| **Corticosteroids** | |  |  |  |  | 0.6722^*^ |
| No | | 13/57 | NE (11.0-NE) | 98.2 (88.0-99.7%) | Reference |  |
| Yes | | 6/21 | NE (8.4-NE) | 100.0 (100.0-100.0%) | 1.23 (0.55-2.78) |  |
| **Anti-epileptic treatment** | |  |  |  |  | 0.4451^*^ |
| No | | 10/36 | NE (10.2-NE) | 97.1 (80.9-99.6%) | Reference |  |
| Yes | | 8/41 | NE (NE-NE) | 100.0 (100.0-100.0%) | 0.70 (0.32-1.52) |  |
| **Age (median = 54 years)** | |  |  |  |  | 0.0208^*^ |
| <=median | | 5/37 | NE (NE-NE) | 100.0 (100.0-100.0%) | Reference |  |
| >median | | 14/41 | 11.0 (9.8-NE) | 97.4 (83.2-99.6%) | 3.14 (1.33-7.41) |  |
| **Initial tumour measurement - length (median= 20.5 mm)*** | |  |  |  |  | 0.5240^*^ |
| <=median | | 2/12 | NE (1.9-NE) | 90.0 (47.3-98.5%) | Reference |  |
| >median | | 4/12 | NE (5.5-NE) | 100.0 (100.0-100.0%) | 1.73 (0.41-7.18) |  |
| **Initial tumour measurement - width (median= 15 mm)*** | |  |  |  |  | 0.1497^*^ |
| <=median | | 1/11 | NE (6.2-NE) | 100.0 (100.0-100.0%) | Reference |  |
| >median | | 5/12 | NE (3.3-NE) | 91.7 (53.9-98.8%) | 4.26 (0.70-25.85) |  |
| **Initial tumour measurement - height (median=11.5 mm)*** | |  |  |  |  | 0.0918^*^ |
| <=median | | 1/11 | NE (6.2-NE) | 100.0 (100.0-100.0%) | Reference |  |
| >median | | 5/11 | NE (3.3-NE) | 90.9 (50.8-98.7%) | 5.24 (0.86-31.87) |  |
| **Product of measurements by DM (median= 2721 mm³)*** | |  |  |  |  | 0.1164^*^ |
| <=median | | 1/11 | NE (6.2-NE) | 100.0 (100.0-100.0%) | Reference |  |
| >median | | 5/11 | NE (3.3-NE) | 90.9 (50.8-98.7%) | 4.76 (0.78-28.86) |  |
| **Intital volume (median = 3126.9 mm³)*** | |  |  |  |  | 0.1164^*^ |
| <=median | | 1/11 | NE (6.2-NE) | 100.0 (100.0-100.0%) | Reference |  |
| >median | | 5/11 | NE (3.3-NE) | 90.9 (50.8-98.7%) | 4.76 (0.78-28.86) |  |
| **Initial maximum diameter (median= 24.5 mm)*** | |  |  |  |  | 0.1150^*^ |
| <=median | | 1/12 | NE (6.2-NE) | 100.0 (100.0-100.0%) | Reference |  |
| >median | | 5/12 | NE (3.3-NE) | 91.7 (53.9-98.8%) | 4.78 (0.79-28.98) |  |
| **NF2 type**** | |  |  |  |  | 0.5192^*^ |
| No | | 5/27 | NE (11.0-NE) | 100.0 (100.0-100.0%) | Reference |  |
| Yes | | 6/26 | NE (10.4-NE) | 96.0 (74.8-99.4%) | 1.47 (0.54-4.00) |  |
| **1p loss**** | |  |  |  |  | 0.4555^*^ |
| No | | 2/15 | NE (11.0-NE) | 100.0 (100.0-100.0%) | Reference |  |
| Yes | | 6/23 | NE (10.3-NE) | 100.0 (100.0-100.0%) | 1.83 (0.47-7.01) |  |
| **22q loss**** | |  |  |  |  | 0.8586^*^ |
| No | | 2/9 | NE (7.4-NE) | 100.0 (100.0-100.0%) | Reference |  |
| Yes | | 6/29 | NE (11.0-NE) | 100.0 (100.0-100.0%) | 0.86 (0.23-3.32) |  |
| **CDKN2AB**** | |  |  |  |  | <.0001^*^ |
| Balanced | | 5/35 | NE (NE-NE) | 100.0 (100.0-100.0%) | Reference |  |
| Deletion | | 3/3 | 5.4 (3.9-NE) | 100.0 (100.0-100.0%) | 25.61 (5.56-118.04) |  |
| **Meningioma class family**** | |  |  |  |  | 0.0013^*^ |
| benign | | 1/10 | NE (10.3-NE) | 100.0 (100.0-100.0%) | Reference |  |
| Intermediate | | 2/17 | NE (10.4-NE) | 100.0 (100.0-100.0%) | 1.29 (0.17-9.72) |  |
| Malignant | | 5/11 | 10.2 (3.9-NE) | 100.0 (100.0-100.0%) | 13.21 (2.04-85.38) |  |
|  | ^KM^Kaplan-Meier method; ^Cox^Cox model; ^*^Logrank test; | | | | | |

Note: Significant associations (p<0.10) are shaded in grey. * Initial measurements analyses including the assessment of the median were performed in the patients with residual disease and available measurement data (n=24). Clinical data were assessed in the ITT population (n=78). **Molecular data were assessed in available tissues of patients consenting to biological research (n=53 for NF2 type and n=38 for 1p loss,22q loss, CDKN2AB, Meningioma class). AKT1 (1 mutated), BAP1 (1 mutated), TERTp (2 mutated), TRAF7 had very small number of molecular events and were not assessed.

**Suppl. Table 7**: Multivariate testing for OS

| **Parameter** |  | **DF** | **Parameter Estimate** | **Standard Error** | **Chi-Square** | **Pr > ChiSq** | **Hazard Ratio** | **90% Hazard Ratio Confidence Limits** | |  |
| --- | --- | --- | --- | --- | --- | --- | --- | --- | --- | --- |
| **Performance status** | **1** | 1 | -16.28914 | 3177 | 0.0000 | 0.9959 | 0.000 | 0.000 | . | |
|  | **2** | 1 | -0.43838 | 1.24140 | 0.1247 | 0.7240 | 0.645 | 0.084 | 4.971 | |
| **Sex** | **male** | 1 | 1.13275 | 1.18496 | 0.9138 | 0.3391 | 3.104 | 0.442 | 21.798 | |
| **Simpsons’ grade** | **4-5** | 1 | 1.81347 | 1.28463 | 1.9928 | 0.1580 | 6.132 | 0.741 | 50.729 | |
| **Age** | **>median** | 1 | 1.56259 | 1.08257 | 2.0834 | 0.1489 | 4.771 | 0.804 | 28.311 | |
| **Meningioma class** | **intermediate** | 1 | -0.45299 | 1.44532 | 0.0982 | 0.7540 | 0.636 | 0.059 | 6.851 | |
|  | **malignant** | 1 | 1.94692 | 1.29622 | 2.2560 | 0.1331 | 7.007 | 0.831 | 59.086 | |
